# A retrospective analysis of ethnic and gender differences in alcohol consumption among emergency department patients: a cross-sectional study

**DOI:** 10.1186/s12873-015-0050-5

**Published:** 2015-09-29

**Authors:** Shahram Lotfipour, Victor Cisneros, Uzor C. Ogbu, Christopher Eric McCoy, Cristobal Barrios, Craig L. Anderson, Wirachin Hoonpongsimanont, Kristin Alix, Bharath Chakravarthy

**Affiliations:** Department of Emergency Medicine, University of California, Irvine Health Affairs, Irvine, 333 The City Blvd West, Suit 640, Orange, CA 92868 USA; Department of Surgery, University of Orange, Orange, CA USA

**Keywords:** Alcohol use, Epidemiology, Gender, Screening, Emergency department, Race, Ethnicity

## Abstract

**Background:**

Previous studies of alcohol use have recognized several trends in consumption patterns among gender and age yet few have examined ethnic differences. This study examines the intra- and inter-ethnic differences in alcohol consumption among a population of patients seen in the emergency department.

**Methods:**

This is a cross-sectional study conducted in the emergency department in a large urban setting. Information on drinking behavior and ethnicity was collected using the Computerized Alcohol Screening and Brief Intervention (CASI) tool. We explored differences in drinking patterns using a multivariate multinomial logistic regression model.

**Results:**

We analyzed the drinking habits of 2,444 patients surveyed between November 2012 and May 2014. The results indicate that when compared to non-Hispanic whites, Asians have the lowest odds of drinking within normal limits or excessively, followed by other Latinos, and Mexicans. Age and gender consistently showed statistically significant associations with alcohol-use. The odds of drinking within normal limits or excessively are inversely associated with age and were lower among females. The predicted probabilities show a marked gender-specific difference in alcohol use both between and within ethnic/racial groups. They also highlight an age-related convergence in alcohol use between men and women within ethnic groups.

**Discussion:**

The results of this study show intra-racial/ethnic variability associated with sex and education. The highlighted differences within and between ethnic groups reinforce the need to use refined categories when examining alcohol use among minorities.

**Conclusion:**

The results of this study confirm some alcohol consumption trends among ethnic minorities observed in literature. It provides empirical evidence of the marked gender differences and highlights an age-related convergence for gender-specific alcohol use. Health-care personnel should be aware of these differences when screening and counseling.

## Background

Alcohol is the world’s largest risk factor for premature mortality, disability, and loss of health, and results in 2.5 million deaths every year [1]. In the United States, excessive alcohol use is one of the leading causes of preventable death, chronic disease and injury, causing a wide range of health and social problems [2]. In 2006, the cost of excessive alcohol consumption, including health care and lost productivity, was estimated at $223.5 billion [3]. Alcohol-related injuries are particularly evident in patients presenting in the emergency department (ED) and trauma centers. It has been estimated that about 10–18% of injured patients in the ED are alcohol-related cases [4]. The range and multitude of alcohol-related injuries seen in EDs and trauma centers makes this an ideal setting for early intervention among patients suffering short-term effects of alcohol, and referral to treatment for patients with long-term alcohol use [5–10].

Studies of alcohol use and alcohol-related injuries have identified several gender and age-related trends in consumption patterns [6, 7]. Men and older adults are also more likely to report higher rates of alcohol consumption. Alcohol-related injuries are more likely to occur in males and young adults when compared to females and older adults. A smaller set of studies have examined the trends associated with ethnic differences in drinking. In 1984, the National Alcohol Survey was one of the first studies that incorporated ethnic minorities and found greater risk of alcohol use disorders among Whites and Native Americans, and greater consequences for Native American, Hispanics, and Blacks [11]. Subsequently studies have identified ethnic-related differences in alcohol consumption and an outsized increased risk of alcohol-related injury or morbidity among Native American, Blacks, and Hispanics [12]. Caetano and colleagues have highlighted that gaps in our literature and the need for further research concerning alcohol behavior among ethnicities, specifically the age and gender among the Hispanic population [13]. This has underscored the need to examine and highlight variations in the risk of excessive alcohol consumption between and within ethnic and racial groups [11–16]. The ED has been identified as an ideal setting for alcohol use screening and screening and brief intervention is associated with a reduction in risky alcohol consumption-related behavior [17, 18].

This study aims to describe variability in drinking patterns across and within ethnic groups among patients seen at a level I trauma center in a large urban setting. Using the Computerized Alcohol Screening and Brief Intervention (CASI) tool, we collected sociodemographic and alcohol consumption data from patients seen in the ED. We hypothesize that there will be significant variation in ethnic/racial-, age-, and gender-specific drinking patterns.

## Methods

This is a cross-sectional study conducted in the university tertiary care ED. The ED is a located in a large urban setting and provides services to inhabitants of the surrounding areas. It is the only level I trauma center in Orange County and handles nearly 40,000 patient visits annually. The Institutional Review Board of the University of California, Irvine, approved this study.

### Study population

We recruited emergency department patients visiting a level I trauma center to participate in this study. Patients were eligible for inclusion in the study if they were i) adults age 18 and over; ii) medically stable; iii) not in custody or on a psychiatric hold; iv) not suspected of being intoxicated; v) English- or Spanish-speaking. The race and ethnicity questions were implemented in CASI in November 2012. This analysis includes patients screened between November 2012 and May 2014. Patient screening occurred between 8 am and midnight, Monday through Sunday because of the availability of research assistants.

### Alcohol use screening in the emergency department

Patients were approached by trained research assistants to screen on a touchscreen tablet (Motion Computing® by Xplore) using the interactive Computerized Alcohol Screening and brief Intervention (CASI) program. CASI was developed by the Center for Trauma and Injury Prevention Research, University of California Irvine Health Affairs, and has successfully been used to identify and intervene with drinkers in the ED setting [6, 19]. It is available in English and Spanish and is based on the Alcohol Use Disorders Identification Test (AUDIT) [20, 21]. The program collects demographic information responds dynamically to the respondents’ answers about the frequency and nature of their drinking. Data collected include age, gender, preferred language (English or Spanish), level of education, frequency of alcohol consumption, amount of alcohol consumed, drinking patterns, self-control and guilty feelings, and impact of drinking on daily life.

### Classification of alcohol use

The classification of alcohol consumption is based on the World Health Organization’s Alcohol Use Disorder Identification Test (AUDIT) used by the National Institute for Alcohol Abuse and Alcoholism (NIAAA) [21]. Four types of drinkers are identified: i) non-drinker, ii) drinker within recommended limits, iii) at-risk drinker, and iv) likely dependent drinker. At-risk patients are those with an AUDIT score of between 8 and 19, likely dependent-drinkers have an AUDIT score of 20 or higher.

### Race and ethnicity

CASI includes two questions used to identify the race and ethnicity of our respondents. We adopted these questions from the 2010 United States Census [22]. The first question identifies whether a respondent is of Hispanic, Latino, or Spanish origin with those who answer yes able to specify whether they are Mexican, Mexican American, or Chicano; Puerto Rican; Cuban; Salvadoran; Guatemalan; Another Hispanic Latino, or Spanish origin. The second question allowed respondents to mark one or more of the following options: White; Black, African American; American Indian, Alaska Native; Asian Indian; Japanese; Native Hawaiian; Chinese; Korean; Guamanian or Chamorro; Filipino; Vietnamese; Samoan; Other Asian; Other Pacific Islander; or to specify another race. We integrated the answers to these two questions to classify our respondents as (i) Non-Hispanic White; (ii) Mexican; (iii) Other Latino; (iv) Asian; and (v) Non-Hispanic Other.

### Data analysis

In our analysis, we used age as a categorical variable with the following categories, 18–20; 21–24; 25–29; 30–39; 40–49; 50–64; 65 and older. The 25–29 group (the highest RRR for drinking within recommended limits) was the reference category. We included three categories for level of education: Less than High school; High school/Some College; Associate Degree/4-year college degree/Advanced Degree (MD, PhD, DSC etc.). High school/Some College (the largest group) was designated the reference group. Race was divided into five categories as defined above. Non-Hispanic white was designated the reference group. For alcohol use, due to small numbers in the likely dependent group, we combined the last two categories (at-risk and likely dependent) creating three categories of alcohol use: (i) non-drinker; (ii) drinks within recommended limits; (iii) drinks excessively. We used non-drinkers as the reference group.

We tested for crude differences in drinking patterns using the chi-squared rest for independence, Kruskal-Wallis test for ordinal variables, and analysis of variance for continuous variables. When the variation among groups was significant, comparisons between individual groups were assessed using the Sidak multiple-comparison test. We explored differences in drinking patterns using a multivariate multinomial logistic regression model. Our baseline model only included ethnicity as an independent variable. Our final model included ethnicity, age, gender, and level of education. We also tested for interactions between race/ethnicity and level of education, and race/ethnicity and gender. The results are expressed as a Relative Risk Ratio (RRR) and predicted probabilities.

## Results

Our study population included 2,444 patients screened using CASI (approximately 4 % of all emergency patients). A large proportion of our respondents were between 30 and 39 (20 %) and 50–64 (20 %) years old. The mean age was 38 years with mean ages ranging from 34 years among Mexicans to 44 among Non-Hispanic White patients. Almost 56 % of the patients screened were male, and the majority of patients had received high school or some college education (57 %). Thirty-four percent of respondents had received an Associate, Bachelor or advanced degree. Thirty-five percent of our study population indicated that they did not drink. A higher percentage of Asians than non-Hispanic Whites did not drink (*p* < .00005). Thirty-three percent drank monthly or less. Only 7 % of our respondents indicated a drinking frequency of four to seven times per week. Among drinkers, non-Hispanic Whites drank more frequently than Mexicans (*p* = .01) and Asians (*p* < .00005). On an average day, the mean number of drinks consumed by our population was 3.2 with those classified as Mexican consuming the highest (3.7) and Asians the lowest (2.4). Mexicans reported more drinks per day than non-Hispanic Whites (*p* < .0005). Binge drinking was also more common among Mexicans than non-Hispanic Whites (*p* = .0001). The characteristics and alcohol use of the study population are presented in detail in Table 1.

**Table 1:** Background and drinking characteristics by racial/ethnic groups

Characteristic	Non-Hispanic White (*n* = 911)	Mexican (*n* = 786)	Other Latino (*n* = 313)	Asian (*n* = 137)	Non-Hispanic Other (*n* = 297)	Total (*N* = 2444)
Age, n (%)						
18–20	58 (6.4)	98 (12.5)	39 (12.5)	13 (9.5)	24 (8.1)	232 (9.5)
21–24	87 (9.6)	137 (17.4 )	66 (21.1)	20 (14.6)	42 (14.1)	352 (14.4)
25–29	88 (9.7)	138 (12.6)	39 (12.5)	14 (10.2)	38 (12.8)	317 (13.0)
30–39	148 (16.3)	176 (22.4)	53 (16.9)	32 (23.4)	72 (24.2)	481 (19.7)
40–49	170 (18.7)	128 (16.3)	56 (17.9)	23 (16.8)	41 (13.8)	418 (17.1)
50–64	244 (26.8)	86 (10.9)	46 (14.7)	30 (21.9)	59 (19.9)	465 (19.0)
65+	116 (12.7)	23 (2.9)	14 (4.5)	5 (3.7)	21 (7.1)	179 (7.3)
Gender, n (%)						
Male	510 (56.0)	442 (56.2)	186 (59.4)	67 (48.9)	152 (51.2)	1357 (55.5)
Female	401 (44.0)	344 (43.8)	127 (40.6)	70 (51.1)	145 (48.8)	1087 (44.5)
Education						
Less than high school	35 (3.8)	122 (15.5)	56 (17.9)	6 (4.4)	8 (2.7)	227 (9.3)
High School/Some College	470 (51.6)	532 (67.7)	167 (53.4)	65 (47.5)	163 (54.9)	1397 (57.2)
AA/4 year/Advanced Degree	406 (44.6)	132 (16.8)	90 (28.8)	66 (48.2)	126 (42.4)	820 (33.6)
Frequency of drinking						
Never	293 (32.2)	283 (36.0)	113 (36.1)	71 (51.8)	106 (35.7)	866 (35.4)
Monthly or less	279 (30.6)	263 (33.5)	108 (34.5)	44 (32.1)	105 (35.4)	799 (32.7)
1 to 3 times/week	245 (26.9)	203 (25.8)	74 (23.6)	20 (14.6)	63 (21.2)	605 (24.8)
4 to 7 times per week	94 (10.3)	37 (4.7)	18 (5.8)	2 (1.5)	23 (7.7)	174 (7.1)
Drinking history						
Average number of drinks on a given day, mean (SD)	2.9 (2.5)	3.7 (2.8)	3.5 (2.5)	2.4 (2.0)	2.7 (2.2)	3.2 (2.6)
Maximum number of drinks on any occasion in the past month, mean (SD)	5.4 (2.8)	6.4 (2.6)	6.4 (2.8)	4.9 (2.8)	5.2 (2.7)	5.8 (2.8)
Binge drinking^a^	(*n* = 618)	(*n* = 503)	(*n* = 200)	(*n* = 66)	(*n* = 191)	(*n* = 1578)
No	336 (54.4)	208 (41.4)	89 (44.5)	42 (63.6)	110 (57.6)	785 (49.8)
Yes	282 (45.6)	295 (58.7)	111 (55.5)	24 (36.4)	81 (42.4)	793 (50.2)

^a^The numbers included here are based on those who indicate that they drink

Table 2 shows the categorization of alcohol use across racial/ethnic groups based on their AUDIT scores. Asians had the highest proportion of non-drinkers (52 %) and the lowest proportion of those drinking excessively (10 %). Non-Hispanic Whites had the highest proportion of those who drink within recommended limits (49 %) and Mexicans and Other Latinos had the highest proportion of those drinking above recommended limits (25 %). Asian respondents had the highest proportion of non-drinkers (52 %) with other ethnic groups ranging from 32 to 36 %.

**Table 2:** Categorization of alcohol use based on AUDIT scores by race/ethnicity

	Non-Hispanic White (*n* = 911)	Mexican (*n* = 786)	Other Latino (*n* = 313)	Asian (*n* = 137)	Non-Hispanic Other (*n* = 297)	Total (*N* = 2444)
AUDIT classification						
Never drinks, n (%)	293 (32.2)	283 (36.0)	113 (36.1)	71 (51.8)	106 (35.7)	866 (35.4)
Drinks with recommended limits, n (%)	444 (48.7)	308 (39.2)	121 (38.7)	53 (38.7)	137 (46.1)	1063 (43.5)
Drinks above recommended limits, n (%)	174 (19.1)	195 (24.8)	79 (25.2)	13 (9.5)	54 (18.2)	515 (21.1)

Table 3 shows the results of the multinomial logistic regression model examining the relationship between race/ethnicity and the modified AUDIT classification among the study population. Non-drinkers were used as the reference model for the analysis, thus we compared non-drinkers and drinking within recommended limits, and non-drinkers and drinking excessively. Age and gender consistently showed statistically significant associations with alcohol-use in the multinomial model. The odds of drinking within normal limits or excessively decrease as age increases and were lower among females than males. Educational attainment was only significant in the model that compared non-drinkers and those who drink within recommended limits. In this comparison, the odds of drinking within normal limits increased as the level of education increased. When comparing non-drinkers to those who drink within recommended limits, the odds of each of the ethnic minorities belonging to this class decrease in comparison to non-Hispanic whites. The most significant decrease in odds is observed among the Asian ethnic group (RRR 0.43; 95 % CI 0.29–0.64). The smallest decrease occurred among the Mexican ethnic group (RRR 0.71; 95 % CI 0.56–0.90). However, when comparing non-drinkers to excessive drinkers only the Asian ethnic group showed a statistically significant reduction in the odds of being a member of that group (RRR 0.26; 95 % CI (0.14–0.50). None of the interaction terms (race/ethnicity and gender; race/ethnicity and level of education) tested in each model were significant.

**Table 3:** Multinomial logistic regression analysis showing the relationship between race/ethnicity and alcohol use

	RRR (95% CI)			
	Unadjusted	Adjusted	Unadjusted	Adjusted
	Non-Drinkers vs Drinks within recommended limits	Non-Drinkers vs Drinks within recommended limits	Non-Drinkers vs Drinks above recommended limits	Non-Drinkers vs Drinks above recommended limits
Race/Ethnicity				
Non-Hispanic White	1.00 (reference)	1.00 (reference)	1.00 (reference)	1.00 (reference)
Mexican	0.72 (0.58–0.89)*	0.71 (0.56–0.90)*	1.16 (0.89–1.51)	0.90 (0.68–1.21)
Other Latino	0.71 (0.53–0.95)*	0.69 (0.50–0.94)*	1.18 (0.84–1.66)	0.93 (0.65–1.34)
Asian	0.49 (0.34–0.72)*	0.43 (0.29–0.64)*	0.31 (0.17–0.57)*	0.26 (0.14–0.50)*
Non-Hispanic Other	0.85 (0.64–1.14)	0.78 (0.58–1.06)	0.86 (0.59–1.25)	0.77 (0.52–1.14)
Age, n (%)				
18–20		0.50 (0.34–0.76)*		0.56 (0.35–0.89)*
21–24		0.97 (0.66–1.14)		1.13 (0.74–1.74)
25–29		1.00 (reference)		1.00 (reference)
30–39		0.63 (0.45–0.88)*		0.65 (0.44–0.98)*
40–49		0.66 (0.46–0.93)*		0.58 (0.38–0.88)*
50–64		0.43 (0.31–0.61)*		0.36 (0.24–0.55)*
65+		0.31 (0.20–0.47)*		0.23 (0.13–0.41)*
Gender, n (%)				
Male		1.00 (reference)		1.00 (reference)
Female		0.75 (0.62–0.90)*		0.38 (0.30–0.48)*
Education				
Less than high school		0.56 (0.40–0.79)*		0.92 (0.63–1.32)
High School/ Some College		1.00 (reference)		1.00 (reference)
AA/4 year/Advanced Degree		1.52 (1.24–1.88)*		1.02 (0.78–1.33)

*Abbreviations*: *RRR* relative risk ratio; *CI* confidence interval; *AA* associate degree

*p value <0.05

**Fig. 1:**
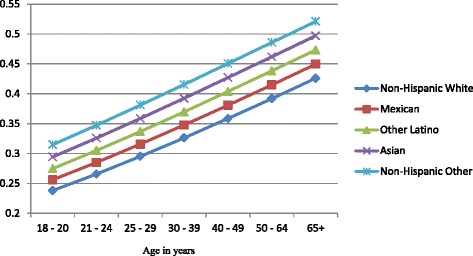
Probability of being a nondrinker across ethnic groups and age categories

**Fig. 2:**
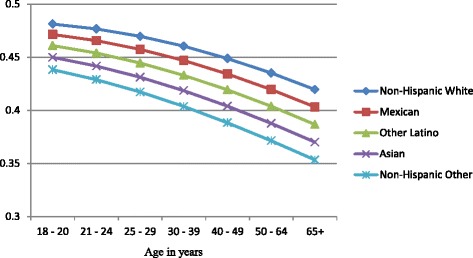
Probability of drinking within normal limits across ethnic groups and age categories

**Fig. 3:**
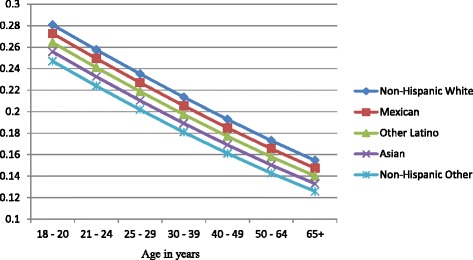
Probability of excessive drinking across ethnic groups and age categories

Figures 1, 2, and 3 show the predicted probabilities of members of each ethnic group being a non-drinker, drinker within recommended limits, or excessive drinker respectively, across the different age groups. The relative rank of each ethnic group was consistent across each alcohol use group with non-Hispanic other and Asians being the least likely to engage in drinking, followed by other Latinos, Mexicans, and non-Hispanic whites in order of increasing likelihood. The probability of being a non-drinker increased with age with those classified as non-Hispanic other or Asian having the highest probabilities in all age groups. Non-Hispanic Whites had the lowest probability of being a non-drinker in all age groups (Fig. 1). Among those who drank within normal limits, there was a gradual decline in the probability of belonging to this group as age increased (Fig. 2). Those classified as Asian and non-Hispanic other showed a slightly larger decline in the probability as age increased. All ethnic groups showed a gradual decline in the probability of being an excessive drinker as age increased at approximately the same rate, with those classified as Asian and non-Hispanic other being the least likely to engage in excessive drinking across all age groups (Fig. 3).

**Fig. 4:**
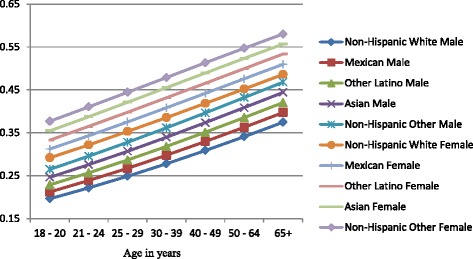
Gender-specific probability of being a nondrinker across ethnic groups and age categories

**Fig. 5:**
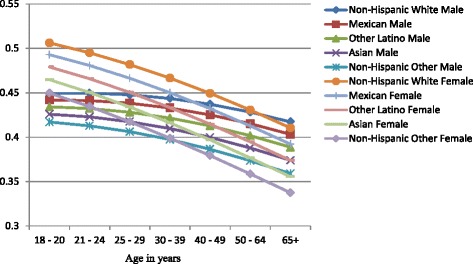
Gender-specific probability of drinking within normal limits across ethnic groups and age categories

**Fig. 6:**
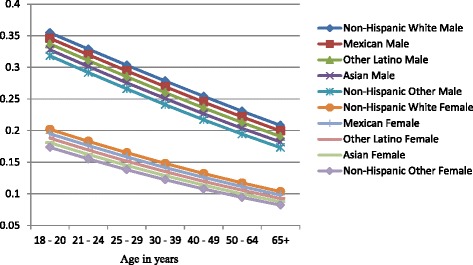
Gender-specific probability of excessive drinking across ethnic groups and age categories

Figures 4, 5, and 6 show gender-specific differences in the predicted probability of members of each ethnic group belonging to each of the alcohol use categories. Among non-drinkers, females in all ethnic groups were less likely to drink when compared to their male counterparts across all ages (Fig. 4). The general ranking pattern noted above was also observed among male and female non-drinkers. Figure 5 shows an age-related convergence in the probability of drinking alcohol within recommended limits. Between the ages of 18 and 20, non-Hispanic white females had the highest probability of belonging to this group, and non-Hispanic other the least likelihood. By age 65, Non-Hispanic white males were the most likely to belong to this group, and non-Hispanic other females the least likely. Overall, young females had a higher likelihood of drinking within recommended limits when compared to their male counterparts but by age 40, a significant reduction in intra-ethnic gender variability is observed. Among excessive drinkers, there is a marked dichotomy in the predicted probabilities of being a member of this group (Fig. 6). At all ages, males are more likely to engage in excessive drinking behavior than females. There is a gradual a decline in the probability as age increases with a slightly larger slope among males than females. By age 65, the probability of engaging in excessive drinking for males is similar to that of females at age 18–20.

## Discussion

We observed ethnic, gender and age-specific differences in the alcohol consumption patterns. Asian respondents were consistently the least likely to engage in drinking behavior, and non-Hispanic white respondents the most likely. Drinking declines with age but at different rates between males and females. The odds of alcohol consumption appear to peak between the ages of 21 and 29, plateau between 30 and 49 and declines from age 50 onwards. This is in keeping with general assumptions and evidence about alcohol consumption during the life course. When compared to men, women have consistently lower odds of drinking either within recommended limits or excessively and higher odds of not drinking. Educational attainment appears to show a linear relationship with the odds of drinking within normal limits. Among the middle-aged and the elderly who drink within normal limits, the age-, gender, and ethnic-differences observed varied significantly.

The multinomial logistic regression model shows that when compared to non-Hispanic whites, Asians have the lowest odds of drinking within normal limits or excessively, followed by Other Latino’s, and Mexicans. This order, non-Hispanics Whites, Mexicans, Other Latinos, Asians, non-Hispanic Other, persisted throughout the study. This relative ranking of the ethnic group remains the same across the age categories, with non-Hispanic Whites most likely to engage in drinking. The differences in the probability of drinking between the ethnic groups narrow as behavior progresses from not-drinking to drinking within recommended limits and excessive drinking. The observed pattern mirrors earlier studies of the impact of age and ethnicity on drinking behavior.

Women of any racial/ethnic group have a higher probability of being a non-drinker than men of any racial group. This increases and persists through all age groups. These gender differences in probabilities are more prominent when examining the likelihood of engaging in excessive drinking. The probability of a men engaging in drinking is almost one and a half that of women for each ethnic group in the early ages and about twice that of women by age 65. While Fig. 5 demonstrates an age-related convergence between males and females, the relative ranking of the ethnic groups remains the same, with non-Hispanic whites the most likely and Asians the least likely. By age 65, non-Hispanic whites (male and female) have the highest probability of drinking within recommended limits, followed by Mexicans (male and female), Other Latinos (male and female), Asian male, non-Hispanic males, Asian females, and non-Hispanic other females. A recent study by Wilsnack et al. of multinational gender differences in drinking noted similar variation in age-related declines in routine/recommended and excessive drinking [23]. They and other authors highlight the impact of sociocultural norms as factor in determining drinking behavior. Among minorities in the United States, these sociocultural factors will be modified over time by acculturation [24]. Thus, there are likely to be further differences between U.S. born and foreign-born minorities. Recent studies have noted increased alcohol use among subsequent generations of migrant women and decreased alcohol use among migrant men because of acculturation [24, 25].

The existing literature has used education as a proxy for socioeconomic status. Studies have primarily aimed at examining the impact of educational attainment on the risk of alcohol dependence [26–28]. The impact is unclear, as some have cited a potentially moderating effect of education while others have noted the correlation between ethnicity and level of education [27, 28]. This study has insufficient numbers of respondents who are likely dependent on alcohol to detect a significant association with education. However, the association with alcohol use (drinking within normal limits) appears to be linear. The odds of using alcohol decrease among those who completed less than high school education when compared to those who have a high school degree or some college. The odds increase among those who have an associate, bachelor or other advanced degree. A study by Satre et al. observed a similar relationship between educational attainment and binge drinking [29]. Blazer and Wu found additional gender variation in drinking habits associated with educational attainment [30].

### Limitations

We collected data from 8 am to midnight because of the availability of research assistants. Patients arrive between midnight and 8 am may have different drinking patterns. Patients were approached for screening while they were intoxicated, but they could be approached in their stay later if sober.

The study relies on self-reported ethnicity and racial classifications and drinking. Self-report for race and ethnic is an advantage over assigned-ethnicity by health-care personnel. We didn’t assess acculturation, and this may influence drinking as well as other behaviors. While it is likely that drinking behavior is under-reported, the anonymous and automated nature of the survey is intended to minimize under-reporting. CASI has been shown to increase detection of alcohol consumption and at risk drinking, as compared to the medical screening exam, in both English and Spanish interviews [19].

Two groups, Native Americans and Blacks, are included in non-Hispanic Other category, as they do not comprise a large proportion of the patient population for the hospital included in this study. However, this does not influence the results for the races included in this study. Other studies have reported difference in age and drinking behavior among Blacks compare other races/ethnic groups. [31] Existing literature suggests that Blacks and Hispanics are less likely than Non-Hispanic whites to use alcohol in their youth. However, age increases rates of alcohol use increases compared to Non-Hispanic whites [31]. Currently our sample size is too small to exam the relationship between Blacks and other ethnicities, this should be revisited in further research. We also have no information about smoking and income-levels, two factors that are associated with drinking behavior. However, the AUDIT score is a validated scale for predicting the risk of injury associated with drinking behavior [32, 33].

## Conclusions

The results of this study confirm some alcohol consumption trends among ethnic minorities observed in literature [14, 16]. It provides empirical evidence of the marked gender differences and highlights an age-related convergence for gender-specific alcohol use. It highlights similarities and differences within the Latino community (Mexicans vs. Other Latinos) and reinforces the need to use refined categories when examining alcohol use among minorities. Many highly educated patients and Whites drank frequently in small amounts. On the other hand, many Hispanics drank less frequently and in larger amounts. Health-care personnel should be aware of these differences when screening and counseling. Future studies examine the effectiveness of interventions tailored to Hispanics and non-Hispanic Whites.

## Abbreviations

CASI: Computerized alcohol screening and brief intervention
ED: Emergency department
AUDIT: Alcohol use disorders identification test
NIAAA: National institute for alcohol abuse and alcoholism
RRR: Relative risk ratio
CI: Confidence interval
